# Global variation of seismic energy release with oceanic lithosphere age

**DOI:** 10.1038/s41598-020-80475-y

**Published:** 2021-01-12

**Authors:** Nicolás Pinzón, Carlos A. Vargas

**Affiliations:** Department of Geosciences, Universidad Nacional de Colombia, 11001 Bogotá, Colombia

**Keywords:** Geodynamics, Geophysics, Seismology, Tectonics

## Abstract

Variations in Mid Ocean Ridge seismicity with age provide a new tool to understand the thermal evolution of the oceanic lithosphere. The sum of seismic energy released by earthquakes during a time, and for an area, is proportional to its lithospheric age. Asthenospheric temperatures emerge on ridge centers with new crust resulting in high seismic activity; thus, the energy released sum is highest on the young lithosphere and decreases with age. We propose a general model that relates the systematic variation of seismic energy released with the lithospheric age. Our analysis evaluates the main physical factors involved in the changes of energy released sum with the oceanic lithosphere age in MOR systems of different spreading rates. These observations are substantiated based on three cross-sections of the East Pacific Rise, six sections in the Mid Atlantic Ridge, and three profiles in the Central Indian Ridge. Our global model provides an additional tool for understanding tectonic processes, including the effects of seismicity and mid-plate volcanism, and a better understanding of the thermal evolution for the young oceanic lithosphere.

## Introduction

The thermal structure and evolution of the oceanic lithosphere govern different geodynamic processes as a function of age^1,2^. The oceanic lithosphere is considered the upper thermal boundary layer of the mantle convection^3^, defined by the 1200 °C isotherm^4,5^, which is formed at the mid-ocean spreading centers. The evolution of this geotherm is associated with the square root of age^6,7^. Hence, the systematic variation of some physical parameters with age became the primary factor to consider as model for the thermal evolution of the lithosphere. The first models were proposed by realizing that the ocean floor heat flow is highest at mid-ocean ridges (MOR) and decreases with distance^7,8^. In this way, these models predict the variation in bathymetry and heat flow with age to describe how the lithosphere cools as it moves away from the spreading center. We recently proposed a model that describes the variation of seismic energy release sum with age in the East Pacific Rise (EPR)^9^. It is based on the main idea that on MOR systems, there is high seismic activity in the ridge axis^10,11^, and due to more stable and colder conditions of the older lithosphere, it decreases as an asymptotic function^9,12^. It proves that the main factor controlling the seismicity of MOR systems is the thermal structure of the lithosphere^13^, and describes the general shape of the depth curve of 1200 °C isotherm, including the flattening for ages > 2 million years old (Myr)^9^. Its main limitation is the unpredictability of seismic energy sum for ages > 4 Ma, which is thought to be due to local instabilities in remote regions from ridge centers or near to boundaries of other tectonic plates.

The thermal structure at a spreading center is predominantly influenced by two factors: (1) the rate of magma supply, and (2) the efficiency of hydrothermal circulation in removing heat^14^. Thus, the spreading rate dependence of seismicity on MOR’s systems becomes the main constraint to relate seismicity on MOR’s with the seafloor age^15,16^. The EPR is considered a fast-spreading ridge (> 6 cm/year)^17^, where the relatively thin brittle lithosphere produces scarce earthquakes that are extremely difficult to locate with the present OBS networks^18^, then, seismically its dynamic spreading process is a little less known in comparison to slow-spreading centers (< 4 cm/year) such as the Mid-Atlantic Ridge (MAR) or the Central Indian Ridge (CIR), where spreading dynamics enhance seismic activity. In this work, our study area includes environments with different spreading rates. We focus on the northern EPR, whose spreading rate ranges from 4 to 7 cm/year^17^, the MAR, in which case the spreading rates vary from 0.6 to 3 cm/year^11,19^, and the CIR where half-spreading rate range from 0.9 to 3.7 cm/year^20^. Specifically, we focus our main observations in areas of the ridge where a relatively high density of earthquakes has been recorded.

The sum of seismic energy released by earthquakes constitutes an important tool for characterizing geodynamic processes. The sum of seismic energy is proportional to the seismic activity in terms of the moment magnitude and the number of earthquakes recorded in a certain area^17,21^. According to Bird et al. (2013), the corner magnitude of spreading earthquakes is about 5.8, independent of the spreading rate. Nevertheless, the number of earthquake events decreases gradually, where rates are close to 40 mm/year. Then decrease considerably at spreading rates larger than 80 mm/year^22^. In this paper, we demonstrate the variations and contrasts in the sum of seismic energy as a function of lithospheric age between different spreading centers and the main implications on the thermal structure beneath each ridge system. Based on these observations, we propose a general model, with a broader scope for the variation of the sum of seismic energy released with age and cooling of young oceanic lithosphere.

## Methods

### Seismic catalog, age, heat flow, and bathymetry

We examined the seismic catalog from the United States Geological Service (USGS) and the Advanced National Seismic System (ANSS), which can be downloaded via GeoMapApp 3.6.10^23^. The database collects earthquakes of magnitude $4.5 \le {m}_{b}\le 7.0$, located between the years 1960 and 2020 for the EPR, MAR, and CIR. The bathymetry data of MOR’s was requested and provided by the National Oceanic and Atmospheric Administration (NOAA). Lastly, the data for ocean crust age were obtained from the digital model of age, asymmetry, and propagation rates of the oceanic crust carried out by Müller et al.^24^ (see Data availability).

### Transect considerations and energy release sum

**Figure 1:**
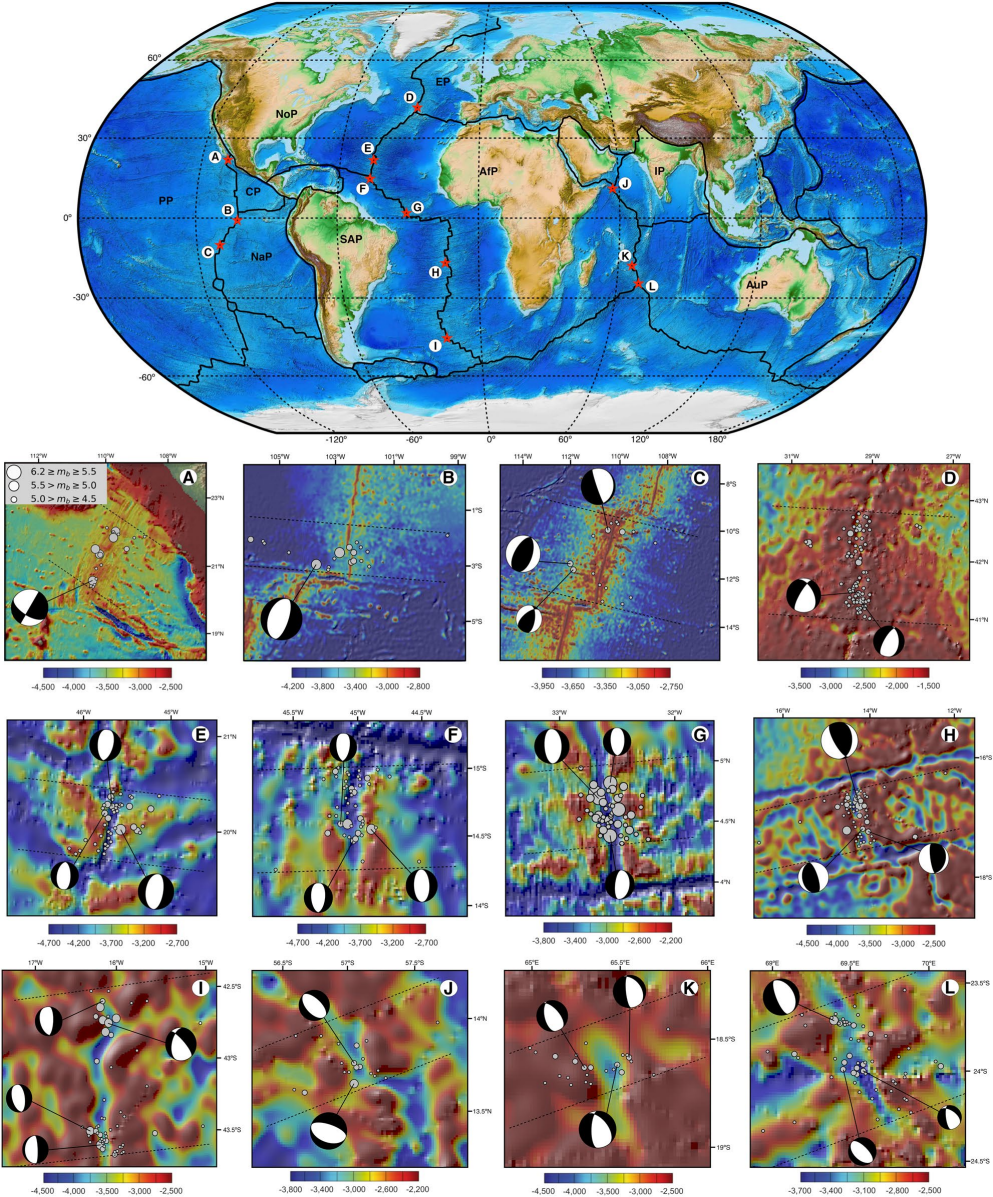
Location of transects. Overview map showing the location of transects (red starts) on different MOR’s systems analyzed in this work. This figure was rendered by using Python 3.8 (https://www.python.org/) and the modulus Basemap 1.0 (https://matplotlib.org/basemap/). Transect (**A**) located between: Pacific Plate(PP)—Rivera Plate (RP); (**B**) Pacific Plate (PP)—Cocos Plate (CP); (**C**) Pacific Plate—Nazca Plate (NaP); (**D**) North American Plate (NoP)—Eurasian Plate (EP); (**E**) North American Plate—African Plate (AfP); (**F**–**I**) South American Plate (SAP)—African Plate (AfP); (**J**–**L**) African Plate—Australian Plate (AuP). The solid black lines show the plate boundaries and dashed black lines shows the corridor. The gray circles represent the distribution of earthquakes and the size depends on the magnitude of the events (${m}_{b}\ge 4.5)$. The earthquake data (1960–2020) were taken from GeoMapApp 3.6.1 (http://www.geomapapp.org/). The bathymetry data was taken from the National Oceanic and Atmospheric Administration (https://maps.ngdc.noaa.gov/viewers/bathymetry) and it is represented by the color bars. The focal mechanisms of events were taken from the Global Centroid Moment Tensor solution catalog (https://www.globalcmt.org/).

We derived twelve perpendicular transects in different MOR areas, three transects located on the EPR, six on the MAR, and three on the CIR (Fig. 1). We selected areas with a special high density of earthquakes recorded in the ridge area located towards the flanks and ridge areas whose transforming boundaries were relatively spaced. Hence, areas with very few events and/or with significantly affected by transform boundaries were avoided. The transect longitudes, and the corridors, depended on the distribution and scatter of events, and longitudes vary between 3° to 5° (approx. 350–550 km), and the transect corridor widths range from 0.3º to 1°. Additionally, due to the fact that some study areas were located close to transforming zones, it was necessary to check the focal mechanisms to exclude the strike-slip mechanism and only to consider earthquakes associated with spreading centers. Also, the corridor is broad enough to prevent the relocation process. We divide the ocean basins into age intervals of 1 Ma, and then we computed the sum of seismic energy released by earthquakes located within each age interval along the transects.

### Seismic energy calculations and global model

The seismic catalog used in this work has limited magnitude resolutions. It is due to the location of seismic networks and limitations for deployments of ocean-bottom seismometers. Hence, the computation of seismic energy released by earthquakes, for each transect, would be restricted to the completeness magnitude (M_c_), which varies from 4.8 to 4.5. Furthermore, the transect with the most number of events involved (Fig. 1D,L) has almost 70 earthquakes. Thus the low number of events try to estimate makes it challenging to use the b-value parameter for evaluating the number of earthquakes below the M_c_ for each age interval. Then, it is important to note that we are not considering the energy released by microseismicity. However, we consider that the contribution of microseismicity to empirical relationships is not significant. Since the sum of seismic energy is calculated using the moment magnitude (M_o_) formula, in this work, we use a series of empirical relationships to obtain the seismic energy released. First, knowing the body-wave magnitude (*m*_*b*_) from the seismic catalog, we used the empirical approach given by Shapira and Hofstetter^25^ to estimate M_o_ from *m*_*b*_ (Eq. 1)_._

1 $$Log\left( {M_{O} } \right) = 1.59m_{b} + 15.63$$

Then we used the model proposed by Hofstetter and Shapira^26^ that relates the seismic energy with M_o_ (Eq. 2), where M_o_ is expressed in dyn⋅cm, and *Log (E*_*O*_*)* is in ergs.

2 $$Log\left({E}_{O}\right)=1.19{log(M}_{o})-8.81$$

Once we computed the sum of seismic energy released for the age intervals along the twelve transects, we performed an average energy release sum for EPR, MAR, and CIR (Fig. 5). Finally, following the methodology proposed by Sclater et al.^8^ and Stein & Stein^1^, we estimated a general relationship between the sum of seismic energy released and the oceanic lithosphere age based on a linear regression by least-squares fit between the binary combination of these parameters.

## Results

### MOR systems and seismic energy released

**Figure 2:**
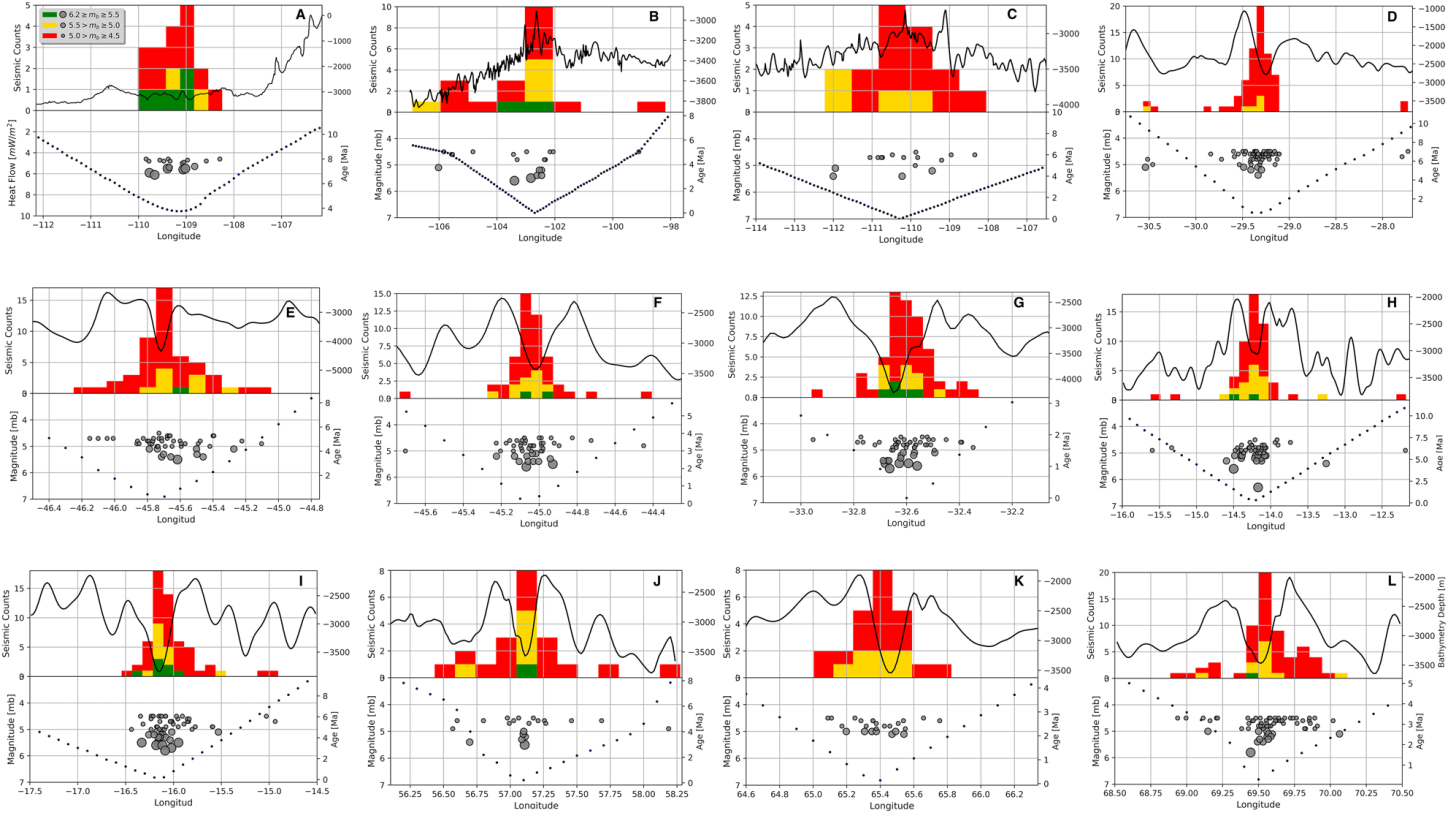
Number and distributions of seismic events, bathymetry, and age in the MOR`s. The bars have been colored according to ranges of magnitude. In the upper part the solid black line represents the bathymetry relief in m.b.s.l. The dotted line at the bottom shows the age of the oceanic lithosphere. The gray circles represent the distribution of earthquakes and the size depends on the magnitude of the events. (**A**–**C**) correspond to three profiles on the East Pacific Rise. (**D**–**I**) correspond to six transects located on the Mid-Atlantic Ridge. (**J**–**L**) correspond to three profiles located on the Central Indian Ridge. This Figure was rendered by using Python 3.8 and the modulus matplotlib 3.3.2 (https://matplotlib.org/).

It is noticeable in the transects that, independent of the spreading rate, there are more seismic events along the ridge axis. The event count decreases towards the axial flanks. Also, scarce earthquakes of high and intermediate magnitude occur preferentially in the young oceanic lithosphere (Fig. 2). Interestingly, we observe that as the spreading rates increase, the number of spreading earthquakes reduces. In contrast, in slow-spreading centers like MAR and CIR (Fig. 2D–L), we observed up to twenty events for the lithosphere younger than 1 Ma. In the EPR, for the same age interval, there occurred only five to ten events (Fig. 2A–C). The EPR transect generally shows a higher frequency of seismic events of low and moderate magnitudes (m_b _< 5.5), mostly concentrated on the ridge axes. At the ridge axes, the 1200 °C isotherm emerges, and as the oceanic lithosphere gets colder, the seismicity production decreases.

**Figure 3:**
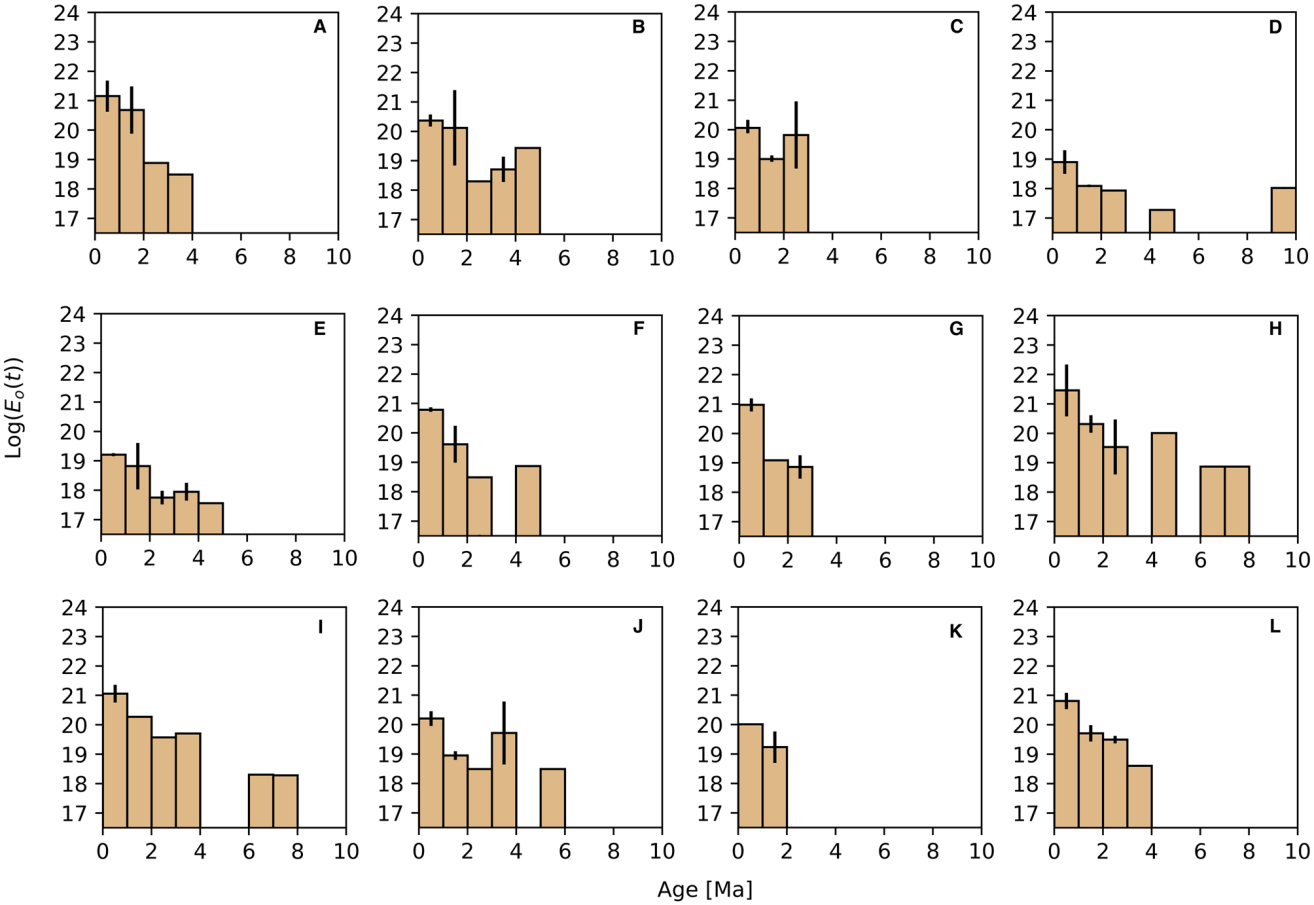
Energy released sum variations with lithosphere age. Bar diagrams show the systematic decrease of the sum of seismic energy released with the oceanic lithosphere age for intervals of 1 Myr with standard deviation bars. (**A**–**C**) age vs the sum of energy released for the three transects on the East Pacific Rise. (**D**–**I**) lithospheric age vs the sum of seismic energy released for the six transects, from north to south respectively, located on the Mid-Atlantic Ridge. (**J**–**L**) age vs the sum of seismic energy released for the three transects on the Central Indian Ridge. This Figure was rendered by using Python 3.8 and modulus NumPy 1.19 for the respective calculations (https://numpy.org/).

**Figure 4:**
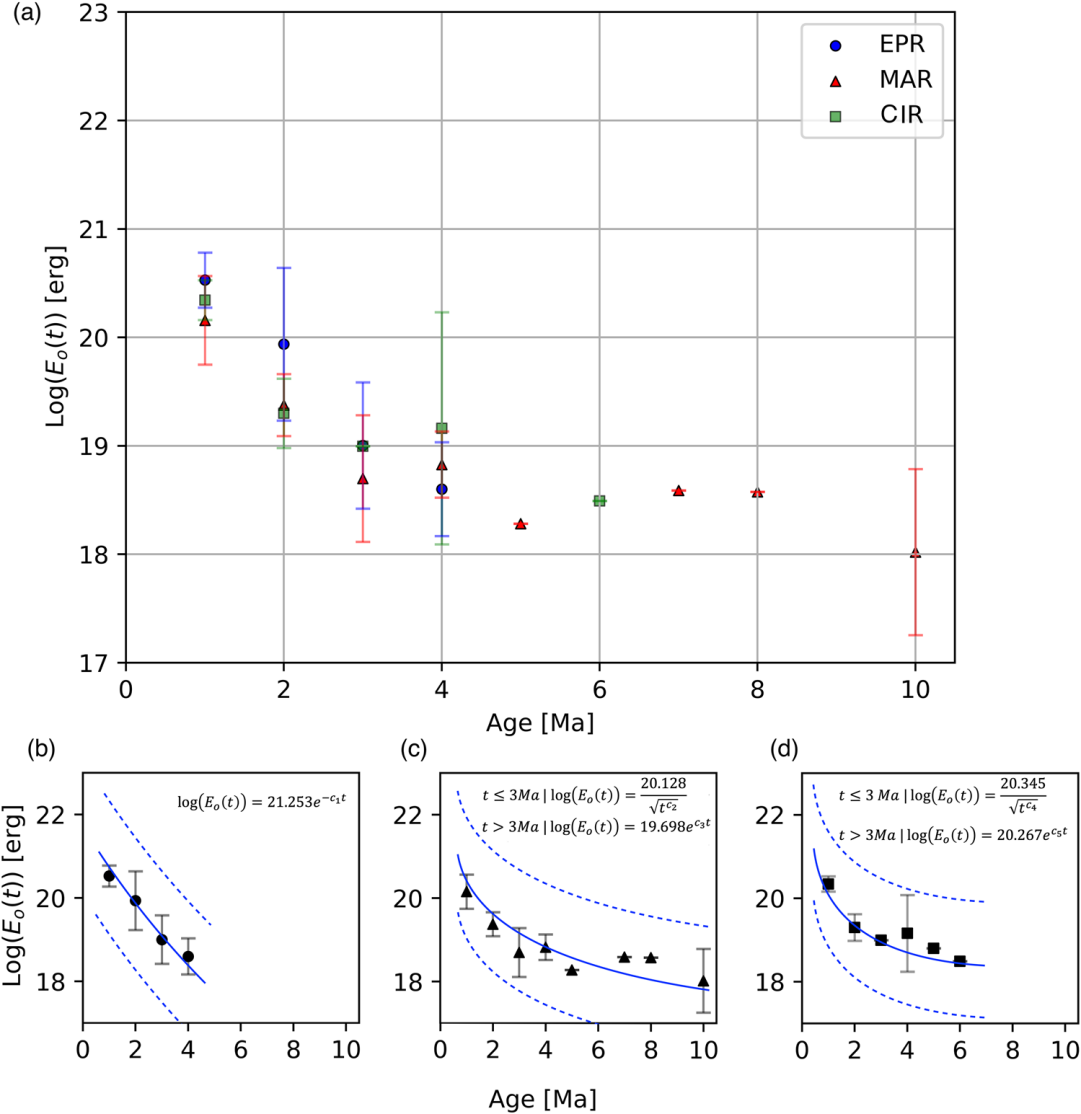
(**a**) Average of energy released sum and standard deviations for different spreading rates. (**b**) empirical relationships between variation of energy released with age for the East Pacific Rise, where ${c}_{1}=-0.034 ;{r}^{2}=0.98$. (**c**) empirical relationships between variation for Mid-Atlantic Ridge, where ${c}_{2}=0.128 ;{c}_{3}=-0.009;{r}^{2}=0.82$. (**d**) empirical relationships for Central Indian Ridge, where ${c}_{4}=0.14 ;{c}_{5}=-0.016;{r}^{2}=0.786$. Dashed blue lines corresponds to confidence interval of 95%. This Figure was rendered by using Python 3.8 and modulus NumPy 1.19 for the respective calculations.

Our results show that changes in spreading rates lead to changes in the seismic regime on MOR’s and, therefore, calculating the sum of energy released for individual transects, we find that it is a little higher in fast-spreading MOR’s systems than for slow-spreading rates regimes (Fig. 3). Also, as the spreading rate decreases, the seismicity has a greater range with respect to the age of the lithosphere. In the EPR (Fig. 3A–C), the sum of energy released decays rapidly with age so that it is only possible to make an approximation at ages no older than 4 to 5 Myr. Instead, for fast-spreading centers like MAR (Fig. 3D–I), the seismic occurrence at older ages allows us to determinate energy values to a maximum limit of 10 Myr. Finally, in the CIR (Fig. 3J–L), our observations show that sum of seismic energy decreases and can be calculated for the lithosphere no older than 6 Myr. On the EPR, we found that the sum of seismic energy released decreases exponentially. However, these observations are restricted to 4 Myr since there are no recorded seismic events at older ages (Fig. 4a). The computed sum of seismic energy released on the youngest lithosphere reaches a value of Log(E_o_) = 20.528 ± 0.253 ergs. It decreases gradually with age to a value of around Log(E_o_) = 18.60 ± 0.432 ergs for the lithosphere not older than 4 Myr. The MAR includes regions with the lowest occurrences of seismic events of moderate to high magnitude (Fig. 2D,E). Therefore, for the first two transects in the north part of MAR, we obtained the lowest values of the sum of energy released (Fig. 3D,E), whereas the energy release values increase in the central and southern part of the ridge (Fig. 3F–I). On the ridge axis, in the lithosphere younger than 1 Myr, we estimated a mean of energy released sum value around Log(E_o_) = 20.399 ± 0.408 ergs, and it decreases steeply until ages around 3 Myr with an energy release value of Log(E_o_) = 18.692 ± 0.583 ergs. In the older lithosphere, between 3 to 10 Myr, the sum of seismic energy released decreases gradually to a value of Log(E_o_) = 18.020 ± 0.765 erg (Fig. 4b). On the CIR, we found that the sum of seismic energy released decrease rapidly for younger ages until 3 Myr approximately.

Moreover, for the lithosphere older than 3 Myr, it decreases more gently until 6 Myr. For the lithosphere younger than 1 Myr, the sum of seismic energy released is Log(E_o_) = 19.984 ± 0.665 erg, and it shows a rapid decay until 3 Myr. For lithosphere ages ranging between 3 and 6 Myr, the sum of energy released decreases gradually since Log(E_o_) = 18.995 ± 0.583 erg to a value of Log(E_o_) = 16.840 ± 0.339 ergs respectively (Fig. 4c).

### Global model

**Figure 5:**
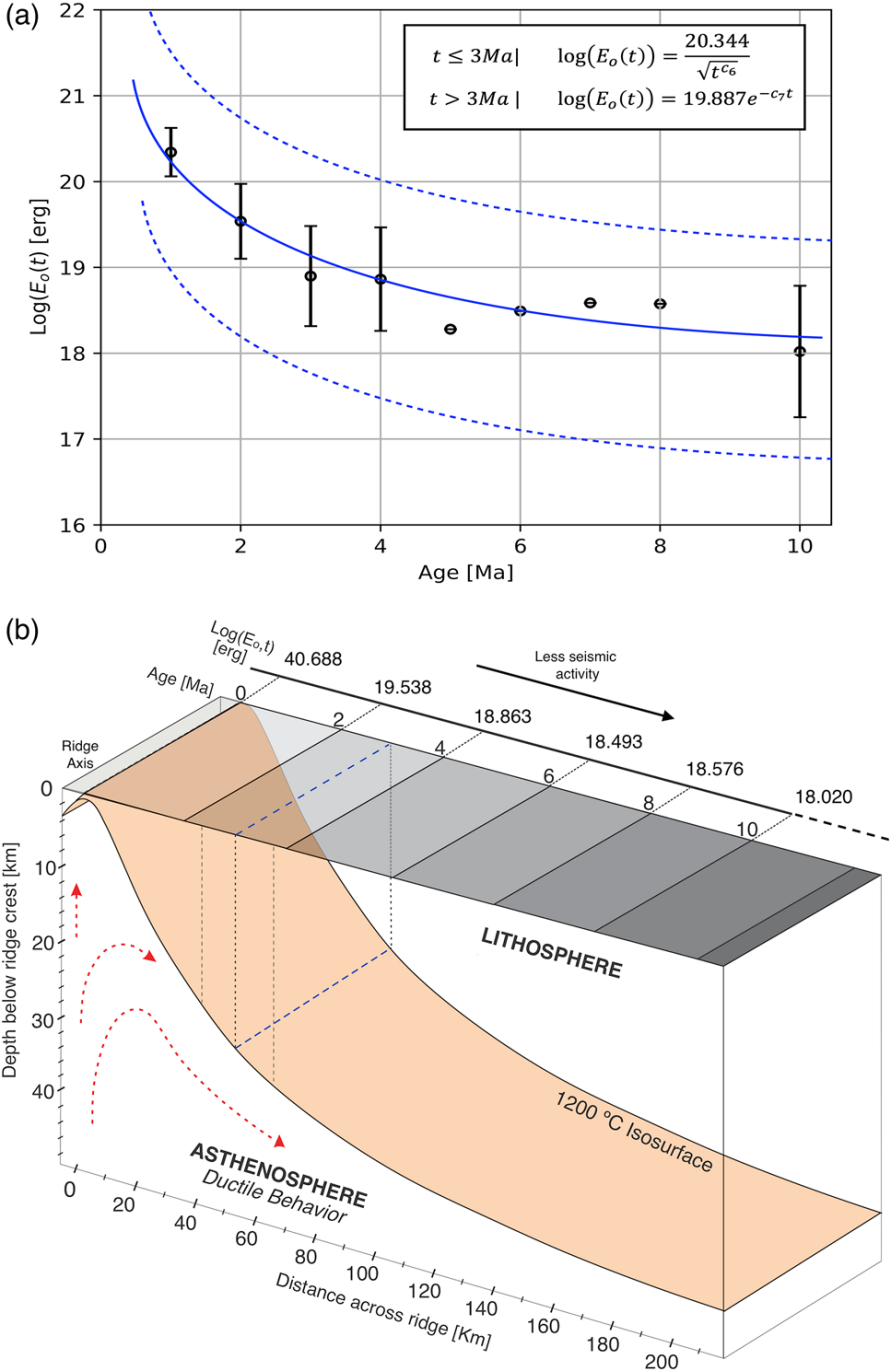
(**a**) Relation between the average of the sum of energy released for all MOR systems and their lithospheric age. The dashed blue line shows the continuous function that best fits the data and is defined according to the formulas in the upper right. The dashed black line represents the confidence intervals (95th percentile). (**b**) Global Conceptual Model for oceanic thermal evolution. The computed sum of seismic energy released as a function of lithosphere age (gray scale). The band around dashed blue line at 3 Ma shows the approximate zone, where hypothetically the 1200 °C isotherm flattening. Dashed red arrows indicate the flow of asthenospheric material. This figure was rendered by using a software for graphic design (https://www.coreldraw.com/la/).

The systematic decrease of the seismic energy released, in the young oceanic lithosphere, from the ridge axis towards the older flanks, is notable in each of the cross-sections made on EPR, MAR, and CIR (Figs. 3, 4). The same behavior according to global bathymetry and heat flow were related to the lithosphere age^1,8,27^. Similarly, we calculated the average of the energy released sum for all MOR systems and proposed a comprehensive model as a function of age and independently of the spreading rate (Fig. 5a). According to our results, the average of the sum of the seismic energy released for a lithosphere no older than 1 Myr is Log(E_o_) = 20.344 ± 0.282 erg. We also found that this energy released decreases rapidly with the square root of age, and it occurs over a lithosphere age of about 3 Myr where the energy released sum is Log(E_o_) = 18.899 ± 0.583 erg. In crusts older than 3 Myr, this relation breaks down, and the energy decreases exponentially to a constant value of Log(E_o_) = 18.02 ± 0.765 erg for lithosphere ages of 10 Myr (Fig. 5b). The statistical adjustment for $t\le 3 Myr$ (see Equations on Fig. 5a) shows a correlation coefficient of $\left|{R}^{2}\right|=0.970$, with a constant value ${K}_{1}=20.344$ and with confidence boundaries of 95% (${K}_{1}[17.795 21.392$]). In a lithosphere with $t>3 Myr$ (see Equations on Fig. 5a), our adjustment shows a correlation coefficient of $\left|{R}^{2}\right|=0.730$, with a constant value ${K}_{2}=19.887$, and confidence boundaries of 95% (${K}_{2}[18.183 18.70$]).

The expressions in Fig. 5a show that the seismic energy released over the axis (t = 0) is infinite. For this reason, we carry out the integration of the formulas to remove the singularity in the proposed system. The limits of the integration extending from ${t}_{i-1}$ to ${t}_{i}$, where ${t}_{0}$ is zero (see Eqs. 3 and 4), and the integral is given in erg⋅Myr. Thus, a finite value for the average energy released in a young lithosphere above the ridge’s axis is found easily.

3 $$t_{i} \le 3{ }Myr{ }\mathop \smallint \limits_{{t_{i - 1} }}^{{t_{i} }} {\text{log}}\left( {E_{o} \left( t \right)} \right) = 40.688{ }\sqrt {\left( {t_{i}^{{c_{6} }} } \right)} { }{-}\sqrt {\left( {t_{i - 1}^{{c_{6} }} } \right)} { }$$

4 $$t_{i - 1} > 3{ }Myr{ }\mathop \smallint \limits_{{t_{i - 1} }}^{{t_{i} }} log\left( {E_{o} \left( t \right)} \right){ } = - 1807.9\left[ {{ }e^{{ - c_{7} \cdot t_{i} }} { }{-}{ }e^{{ - c_{7} \cdot t_{i - 1} }} } \right]$$

where $${c}_{6}=0.126 {Myr}^{-1}$$ and $${c}_{7}=-0.011 {Myr}^{-1}$$.

It is necessary to bear in mind that these expressions are restricted to the seismicity around the ridge. Thus, the main limitation to generate these empirical relations are aseismic zones in MOR’s; hence, we considered only the most seismically active zones. According to the study areas, it was possible to compute the sum of seismic energy for the lithosphere no older to 10 Myr (Fig. 5a). Furthermore, we consider that to generate a more representative model, observations in more ridge systems must be rendered. In that case, the outcome of new scenarios may gradually modify the expressions proposed. furthermore, to the extent that digital signals are used to calculate energy, it is expected to have less uncertainty with respect to the models presented in this work.

## Discussion

The results obtained for each MOR system show a variation of the sum of seismic energy released with the spreading rate. At slower spreading rates, the energy released would be expected to decrease (Fig. 4). However, for fast-spreading rates, the sum of seismic energy released could be computed only for very young oceanic lithospheres (EPR ≤ 4 Myr). Whereas for low spreading rates, it was possible to estimate the energy for slightly older oceanic lithospheres (MAR ≤ 10 Myr). One of the relevant factors that seems to determine the seismicity occurrence in young plates is the high thermal gradient in areas close to the axis of the spreading center^9,28^, which is governed by the isotherm 1200 °C^5^. Thus, we assume that for fast-spreading rates, the isotherm is near the surface and high temperatures due to the a steeper geotherm than on slow-spreading rate areas, where the isotherms would be flatter. These observations can be supported by taking into account some physical variables that have an effect on the behavior of the 1200 °C isotherm. Hydrothermal flow at oceanic spreading centers remove for about ten percent of all heat flux in the oceans and controls the thermal structure of young oceanic plates^2,29^. Additionally, hydrothermal cooling enhances cracking events in the upper crust of ridge environments^30^. Previous hydrothermal circulation studies on MOR’s show deep, but perhaps more significant, heat sources at slower spreading ridges. They provide larger and longer-lived but sparser hydrothermal venting sites and cooling. The steady, near-continuous, shallow magma supply at the fast-spreading ridges leads to frequent but small-scale vent fields^31^. Hence, the upper crust is, therefore, sensitive to crustal inflation and/or heating. Still, another possibility to consider is that seismicity could vary as the stress concentrations change with the shallow heat source of the axial magma chamber^32,33^. If this is so, concentrations of stress near the slow-spreading ridge center would be greater than stresses on fast-spreading rates giving rise to increased seismicity on the last ones. A good way to verify this is through the spatio-temporal monitoring of b-value, and them analyses more careful the state of stress before and after of quakes of magnitude (m_B_) > 4.5. In general, detailed investigation would be required to determine the contributions of these physical factors.

The frequency of the oceanic intraplate earthquakes shows an apparent variation for different spreading rates. The maximum number of events for an interval of less than 1 Myr occurs in slow-spreading centers and this quantity decrease with the spreading rate. On slow-spreading rate centers, such as MAR and CIR, the seismicity involved greater age ranges of the lithosphere than fast-spreading. It could be due to a high thermal gradient, variations in hydrothermal circulations, and increasing extensional stresses in the axial ridge.

Our results show a variation in the sum of seismic energy released with the age of the lithosphere, and globally it can be expressed according to the formulas in Fig. 5. In particular, our model predicts the energy released sum with age for young oceanic lithospheres (t ≤ 10 Myr). We suggest that the variations in the energy released with age are an expression of the thermal evolution of the young oceanic lithosphere. Hence we used the calculated data of energy released sum for the global model of the oceanic thermal evolution (Fig. 5b). Our model suggests that, probably due to a high thermal and highly stressed areas, the sum of energy released decreases steeply on a very young lithosphere (< 3 Myr approx). Thus, we assume that the 1200 °C isotherm has a steep descent to depths close to 30 km for the lithosphere age around 3 Myr. For crust older than 3 Ma, the isosurface could change its trend and begins an asymptotic flattening, where the thermal gradient should be lower and stable, resulting in a low rate of seismicity in the upper crust. The dramatic depth-changes in the general shape of the isosurface, between 2 and 3 Myr, can also be explained by variations in the elastic thickness of the oceanic lithosphere and by the effects of hydrothermal circulation that, together and in a delicate balance, control the thermal gradient of the lithosphere.

## Data Availability

The global earthquake dataset was downloaded freely using the GeoMapApp web application. This tool have databases provided by the United States Geological Survey, and the Advanced National Seismic System, which are also available respectively at https://earthquake.usgs.gov/earthquakes/search/ and http://www.ncedc.org/anss/catalog-search.html. We used the digital model of age, asymmetry, and propagation rates of the oceanic crust carried out by Müller et al. (2008), available at https://www.ngdc.noaa.gov/mgg/ocean_age/ocean_age_2008.html. Finally, the bathymetry data was downloaded from the National Oceanic and Atmospheric Administration (NOAA), available at https://maps.ngdc.noaa.gov/viewers/bathymetry.

## Code availability

The analysis codes and related scripts in Python for generating figures used in the main text are available from the corresponding authors upon reasonable request.
